# “UHAND”—A National Cancer Institute Funded Partnership to Advance Cancer Health Equity through Scholar Training

**DOI:** 10.3390/ijerph18105054

**Published:** 2021-05-11

**Authors:** Arooba A. Haq, Lorraine R. Reitzel, Tzuan A. Chen, Shine Chang, Kamisha H. Escoto, Kayce D. Solari Williams, Crystal Roberson, Litty Koshy, Lorna H. McNeill

**Affiliations:** 1 Department of Psychological, Health, and Learning Sciences, University of Houston, 491 Farish Hall, Houston, TX 77204, USA; aahaq2@central.uh.edu (A.A.H.); tchen3@central.uh.edu (T.A.C.); kdsolari@central.uh.edu (K.D.S.W.); littyk92@gmail.com (L.K.); 2 HEALTH Research Institute, University of Houston, 4849 Calhoun Road, Houston, TX 77204, USA; 3 Department of Epidemiology, University of Texas MD Anderson Cancer Center, 1155 Pressler St., Houston, TX 77030, USA; shinechang@mdanderson.org; 4 Department of Health Disparities Research, University of Texas MD Anderson Cancer Center, 1400 Pressler St., Houston, TX 77030, USA; khescoto@mdanderson.org (K.H.E.); clroberson@mdanderson.org (C.R.); lmcneill@mdanderson.org (L.H.M.)

**Keywords:** UHAND Program, cancer disparities, cancer health equity, women, minorities, educational training program

## Abstract

Black and Hispanic adults are disproportionately affected by cancer incidence and mortality, and experience disparities in cancer relative to their White counterparts in the US. These groups, including women, are underrepresented among scientists in the fields of cancer, cancer disparities, and cancer care. The “UHAND” Program is a partnership between institutions (University of Houston and The University of Texas MD Anderson Cancer Center) aiming to build the capacity of underrepresented and racial/ethnic minority student “scholars” to conduct research on eliminating cancer inequities by reducing social and physical risk factors among at-risk groups. Here, we examine the outcomes of the UHAND Program’s first scholar cohort (n = 1 postdoctoral fellow, n = 3 doctoral scholars, n = 6 undergraduate scholars). Data collection included baseline, mid-program, and exit surveys; program records; and monthly scholar achievement queries. From baseline to exit, scholars significantly increased their research self-efficacy (*p* = 0.0293). Scholars largely met goals for academic products, achieving a combined total of 65 peer-reviewed presentations and nine empirical publications. Eight scholars completed the 2-year program; one undergraduate scholar received her degree early and the postdoctoral fellow accepted a tenure-track position at another university following one year of training. Scholars highly rated UHAND’s programming and their mentors’ competencies in training scholars for research careers. Additionally, we discuss lessons learned that may inform future training programs.

## 1. Introduction

### 1.1. Importance of Workforce Diversity in Cancer Disparities Research

The underrepresentation of women and individuals identifying as members of racial/ethnic minority groups in cancer disparities research and cancer care is well known [1,2], as are the potentially linked racial and ethnic disparities in cancer risk, cancer incidence, and cancer mortality. For example, African American/Black (hereafter, Black) and Hispanic/Latinx (hereafter, Hispanic) men and women bear an unjust burden of incidence and mortality from several cancers (e.g., breast, lung) relative to their non-Hispanic White counterparts [3]. Relatedly, Black and Hispanic adults also experience disparities in cancer risk behaviors, whereby they have greater incidence of physical inactivity, a poorer diet, greater rates of overweight/obesity, and more difficulty quitting tobacco than non-Hispanic White adults [4,5]. Prior studies have found that nearly half of cancer incidence is attributable to environmental and lifestyle factors, with tobacco use, excess body weight, and alcohol intake accounting for 29%, 7%, and 4%, respectively, of approximately 9000 male and 7000 female deaths annually [6,7,8,9]. These are modifiable behaviors/conditions that—if targeted in most at-risk and general populations—may prevent cancer incidences that are not solely hereditary in origin. Moreover, in some cases, women bear more of the burden of cancer risk behaviors relative to men (e.g., higher rates of severe obesity [10], greater smoking relapse rates [11]), making their inclusion in the field critically important to better understanding and addressing these disparities that can ultimately impact cancer incidence and mortality.

There are many benefits of training a diverse workforce to address cancer and cancer risk disparities. For example, minority and women researchers may better understand and address the needs of their communities through their unique perspective and awareness of issues that are critical to solving disparities in their communities [12]. This understanding includes how social determinants, or conditions in the places where people live, learn, work, and play [13,14], affect cancer risk and risk behaviors [15,16,17,18]. Additionally, it is important to have researchers and medical professionals from minority populations involved in cancer research to foster population representativeness in clinical trials to better achieve cancer health equity [17,19]. Moreover, research has shown that diverse teams outperform homogenous ones when working together advantageously on incorporating innovation and distinct perspectives [20]. However, further work is needed to address the current research gaps regarding how the social determinants of health disproportionately contribute to cancer health disparities experienced by Black and Hispanic groups [15], as well as how to develop and prepare a diverse workforce to lead this work.

Recognizing the importance of training minority and women scientists in order to more ideally address cancer health disparities, the National Cancer Institute (NCI) and other funders have invested heavily in the development of research and training programs [21]. However, more programs focusing on cancer behavioral risk factors such as diet, exercise, and weight management, and those specifically dedicated to developing women and racial minority populations for this work are needed [17,19,22,23]. There is also a need for better knowledge dissemination and translation between researchers and diverse communities [24,25]. Knowledge translation is particularly important in prevention as communities that have greater awareness and understanding of healthy lifestyle behaviors to reduce cancer risk are likely to have improved lifestyle behaviors, treatment options, patient advocacy, and patient compliance. Thus, it is extremely important to address these gaps in training programs to provide a more comprehensive approach in addressing cancer disparities with a more diverse workforce.

### 1.2. UHAND Partnership Program

The “UHAND” Program is a collaboration between the University of Houston (UH) and The University of Texas MD Anderson Cancer Center (MDA), funded by the NCI under a Feasibility Studies to Build Collaborative Partnerships in Cancer Research (P20) initiative (PAR-16-084). The purpose of this collaboration is to bring together a minority-serving institution and a comprehensive cancer center to create a comprehensive research/educational training program that would provide opportunities for underrepresented student “scholars” to develop careers in behavioral cancer prevention and cancer disparities research. Although not a requirement of the funding mechanism, both institutions are located close to one another and in Houston, Texas, the 4th largest metropolitan area in the US [26]. The UH is the second most ethnically diverse major research university in the US and is designated as a Hispanic-Serving Institution and an Asian American and Native American Pacific Islander-Serving Institution by the US Department of Education, Office of Postsecondary Education [27]. MDA has been named as one of the top two US cancer hospitals in the U.S. News & World Report’s “Best Hospitals” survey annually since 1990 [28].

**Figure 1:**
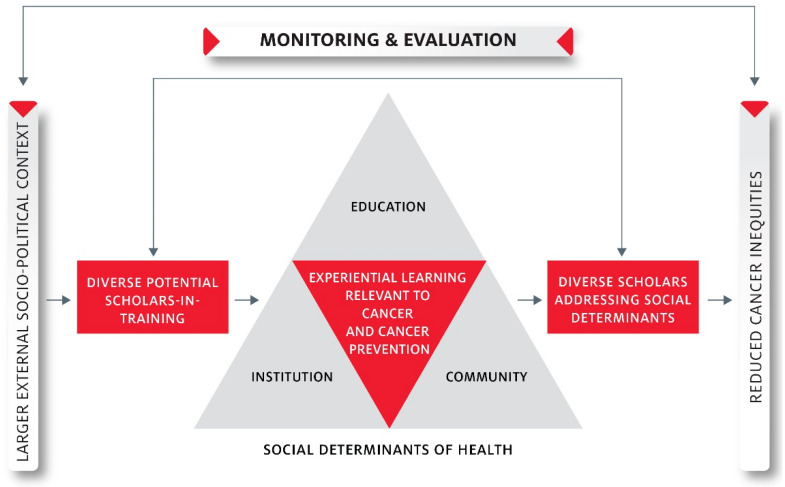
UHAND Program’s Conceptual Model.

The UHAND Program’s overarching goal is to ensure that scholars preparing to join the scientific workforce have the necessary skills and capacity to eliminate cancer inequities through the reduction of social and physical risk factors among disproportionately affected groups. The UHAND Program provides support for developing scholars and early-stage investigators (ESIs) to better understand and conduct intra-institutional research projects that address the social determinants of cancer risk behaviors that predispose Black and Hispanic groups to disproportionate cancer risk. The UHAND Program provides integrated education, broad stakeholder engagement, and targeted approaches to involve women, Hispanic, and Black scholarsin cancer research (see Figure 1). 

The UHAND Program incorporates best practices of successful training programs, such as the use of a variety of learning methods [29,30,31] and the continuous evaluation of data on programmatic impact and outcomes [30]; likewise, it features several relatively unique characteristics, such as collaboration between researchers and advocates [24,25], and between institutions [32]. Additionally, the UHAND Program prioritizes diverse minority researchers and women for participation, focuses on behavioral risk factors for cancer, and includes knowledge dissemination and translation between researchers and community through social media and other means. Altogether, these components and the collaborative partnership resulted in the Program’s funding by the NCI at first submission. 

#### 1.2.1. UHAND’s Major Goals

The UHAND Program has four specific goals. The first goal was to develop a research and educational partnership between the UH, MDA, and local community-based organizations to stimulate collaborative cancer disparities research related to tobacco use, poor diet, and physical inactivity among Black and Hispanic individuals. The second goal was to support the development of ESIs through a rigorous Pilot Research Program that facilitates direct-experience proposing, conducting, and leading cancer disparities research, with the support of a larger UHAND Program and its undergraduate, graduate, and postdoctoral scholars. The third goal was to increase the number of underrepresented racial/ethnic minority scholars and faculty engaged in cancer disparities research by providing them with research training, mentorship, and service-learning experiences. Finally, the fourth goal was to develop a robust community outreach program focused on engaging community members in cancer disparities education, research, and clinical trials.

#### 1.2.2. UHAND Program Components

There are three main components to the UHAND Program: an Education Program, a Pilot Research Program, and a Community Outreach Program.

The Education Program (EP) serves the undergraduate scholars, doctoral scholars (hereafter referred to as graduate scholars), and postdoctoral fellows who represent diverse backgrounds and who were invited to participate in the UHAND Program through a rigorous application and selection process. The EP’s goal is to enable scholars to develop the attitudes, knowledge, and skills necessary to conduct research on reducing cancer disparities, with a focus on lifestyle behaviors (e.g., tobacco use, poor diet, physical inactivity) that increase cancer risk for Black and Hispanic adults. This is done by providing the scholars with mentored research projects, seminars in cancer disparities and career development, interactive and community-based service-learning experiences, and summer research experiences, all guided through individual development plans (specifically myIDP [33], one of two IDPs recommended by the NIH) and executed between scholars and their mentors. Scholars also receive at least 8 h of ethics and responsible conduct of research training. All scholars are paired with university research faculty mentors—from either UH or MDA—who have expertise in cancer risk, social determinants of health, clinical and population cancer research in Black and Hispanic populations, and student mentoring. UHAND Program participation was designed to span a 2-year training period. 

The Pilot Research Program (PRP) is comprised of ESIs who work on pilot projects with senior mentors and an experienced investigative team. The PRP provides support to new investigators in developing innovative and impactful research conducted in community and clinical settings within cancer disparities research, particularly within tobacco- and lifestyle-related disparities research. The UHAND Program grant included funding to support two pilot projects: one on the stress-based biological and behavioral cancer risks among Mexican immigrants, and another on a lifestyle intervention for Black prostate cancer patients on active surveillance and their partners. The PRP was later expanded to include additional projects through Administrative Supplements that extended the scope of the initial two pilot projects. The extended scope incorporated the role of physical activity perceptions and barriers in Mexican immigrant cancer risks, and an evaluation of skeletal muscle strength and function among Black prostate cancer survivors in order to reduce the risk of developing cardiometabolic diseases and ultimately, to improve the quality of life in survivorship. UHAND scholars participated in these projects, assisting with data collection and analyses.

The Community Outreach Program (COP) is integrated in the cancer health equity development of scholars and in the execution of pilot research projects to increase the real-world impact of these efforts. The COP comprises UHAND team members with community experience and hosts UHAND Programmatic events, informs community members about the UHAND Program, coordinates opportunities for scholars to attend health fairs and seminars, provides scholars with opportunities to present research to community representatives for feedback, works with scholars in fostering presentation skills to community audiences, and offers scholars unique opportunities to connect with the local community. The COP includes a Community Partners Network of local health-based organizations that provide service-learning opportunities for scholars, and Community Mentors who work with scholars on mentor plans and meetings, provide research feedback from the community perspective, enable access to at-risk populations, and identify local opportunities for sharing research findings. The COP team also works closely with the Community Advisory Board (CAB, elaborated below). The overall aim of the COP is to enable bi-directional communication between the community and the UHAND scholars to enhance the translation and potential impact of UHAND research.

#### 1.2.3. UHAND Structure and Guiding Boards

The UHAND Program was funded through two grant awards (P20CA221696 and P20CA221697): one given to a Principal Investigator (PI) at MDA, and another to a PI at UH. Additionally, the components of the UHAND Program (e.g., EP) each have co-leadership and support staff from both MDA and UH, facilitating a true partnership between institutions.

The UHAND Program is guided by a CAB, an Internal Advisory Committee (IAC), and an External Advisory Board (EAB). The CAB comprises prominent community leaders across a range of sectors in the greater Houston area that work collaboratively with the UHAND team in providing training, outreach, and research activity support, in addition to providing guidance for the dissemination of project findings in the community. The IAC comprises institutional leaders and researchers with equal representation from UH and MDA, and assists with scholar selection, institutional support, and program sustainment. The EAB comprises nationally renowned researchers and provides broad scientific direction to and drives innovation in the UHAND Program’s research activities, in addition to keeping the UHAND team aware of new and innovative research and educational practices in the cancer disparities field.

### 1.3. The Current Report

The purpose of this report is to present information on the UHAND Program’s first cohort of scholars and lessons learned that may inform similar training programs. Our objectives, delineated more fully in the Methods section, were broadly to enhance the scholars’ research self-efficacy and academic output through competent mentorship and their sustained participation in the UHAND program, which would ultimately lead to interest in, and be an evidence of, the pursuit of higher education (and/or faculty positions) in the cancer disparities field. Our results are presented in relation to these objectives. This work may provide a comprehensive, evidence- and need-based model for other educational training programs with similar goals for improving cancer health inequities by creating more cancer research career opportunities for women and minorities.

## 2. Materials and Methods

### 2.1. Participants and Procedures

Table 1 describes the UHAND Program’s first cohort of scholars. There were 10 scholars (1 postdoctoral fellow, 3 graduate scholars, and 6 undergraduate scholars) actively participating in Cohort 1. The UHAND Program’s first cohort of scholars began in the summer of 2018 and continued in the program for about 2 years until late May 2020.

#### 2.1.1. Scholar Eligibility Criteria

The eligibility criteria for admission to the UHAND Program were the following: (1) undergraduate or doctoral student enrolled in, or recently accepted into, a full-time degree program at the UH; (2) having at least 2 years remaining in their degree plans prior to graduation at the start point of the UHAND programming; (3) to have backgrounds in, or be interested in, behavioral and social sciences, social work, communications, biomedical sciences and related public health disciplines, or any other disciplines relevant to studying cancer health disparities; and (4) to be a US Citizen or permanent resident (a requirement of the funder). The eligibility criteria for the postdoctoral fellow included the need to have successfully earned a doctoral degree by the start point of the UHAND programming, and interests in cancer health disparities complementary to those of the intended mentor. In the case of the 1st UHAND Program cohort, that was the UH PI. It was not a requirement to be underrepresented in the sciences to be eligible for UHAND Program participation, but demographic data were collected at the point of application and women and/or individuals from racial/ethnic groups underrepresented in the sciences were prioritized for admission.

#### 2.1.2. Recruitment of Scholars

Undergraduate and graduate scholars were recruited through the UHAND website, UHAND social media accounts, free-standing displays and recruitment materials at UH events, presentations in UH classes, word of mouth from UH faculty members to students, and interactive UH events such as UH Honors College summer research recruiting event and UH career fairs where UHAND team members presented the program [34]. The postdoctoral fellow was recruited through a national search through a job ad to facilitate a competitive pool of applicants more quickly than a local recruitment-only approach may have allowed. The job posting was shared with colleagues and on professional listservs, and distributed via targeted emails at the partnering institutions.

#### 2.1.3. Selection of Scholars

Undergraduate and graduate scholars were selected from a variety of UH majors through a 3-level review process. First, the UHAND EP screened application materials and ranked candidates. Next, the UHAND IAC reviewed application materials and ranked applicants. Finally, the UHAND PIs reviewed application materials, received and reviewed rankings, and interviewed candidates. Applications were reviewed for demonstrated academic accomplishments, potential for academic and scholarly success (as indicated from letters of recommendation and transcripts), interest in cancer/health/social disparities, and prior research and/or working experiences. All information was considered by the UHAND PIs in making final admission selections.

Out of 5 complete graduate scholar applications and 17 complete undergraduate scholar applications, 3 graduate and 6 undergraduate scholars were invited to join the program. The proportion of undergraduates versus graduate scholars invited to join the first cohort was based on selecting the best candidates of both pools and accounting for the maximum number of scholars we could afford on the budget, balancing that undergraduate inclusion was more affordable than graduate inclusion, based on respective pay rates. As part of their application materials, scholars indicated the top three mentors they would like to work with from a list on UHAND’s website. Potential mentors (n = 10 MDA, n = 9 UH) were selected by the UHAND PIs based on their engagement in social/behavioral cancer disparities work and their agreement to participate. Scholars were matched to top-choice mentors, with consideration for ensuring distribution across potential mentors. All approached mentors (4 from UH and 4 from MDA; some mentored >1 scholar) accepted the scholar as a mentee. Postdoctoral applicants applied to the UH and were interviewed by the UH PI, with whom the selected candidate would directly work. In this case, the successful applicant was not previously associated with either of the partnering institutions.

Undergraduate and graduate scholars were paid an hourly rate and committed 20 h per week to work with their research mentors and participate in the UHAND Programming (i.e., Cancer Disparities Seminars, Cancer Prevention and Control Grand Rounds, Community Presentations, Writing Sessions, Research Webinars, and other seminars). For undergraduate scholars, UHAND participation also included participation in the UH Summer Undergraduate Research Fellowship (SURF) program, where they learned about various aspects of research—from topics in data science and responsible conduct of research, to learning how to develop effective resumes and poster presentations, applying for awards and graduate school, and managing expectations and challenges in research [35]. They were also given an opportunity to formally present their research project results. The postdoctoral fellow was selected and appointed as a full-time scholar in the UHAND program with a designated faculty mentor. She also participated in all UHAND programming.

### 2.2. Scholar Outcome Measures

#### 2.2.1. Research Self-Efficacy (Goal 1)

Surveys with questions on research self-efficacy were administered at baseline (2018), mid-program (2019), and exit (2020). Postdoctoral fellow and graduate scholar responses were combined to facilitate confidentiality. A modified version of Forester et al.’s research self-efficacy scale was used [36]. The original 33 items were slightly modified and reduced to 14 relevant items regarding successfully accomplishing research related tasks (e.g., writing a research paper, collecting data, formulating hypotheses, etc.), with response options ranging from no confidence (0) to total confidence (9). Two additional investigator-generated items asked about interest and likelihood in pursuing a career in cancer disparities research, with response options ranging from not at all (0) to extremely (4). Our explicit goal was to increase research self-efficacy over time among UHAND scholars from baseline to exit. The research self-efficacy scale demonstrated strong reliabilities (2018: 0.92; 2019: 0.95; 2020: 0.91) in this sample.

#### 2.2.2. Academic Products (Goal 2) 

The number and category of academic products were assessed by scholar report throughout the duration of the program. Additionally, an annual survey following program exit captured academic products, noting UHAND support that came to fruition following their enrolled period in the UHAND program. Due to the commonly experienced lag between submission and presentation delivery/publication, our timeframe to assess adherence to our explicit goal was extended to the end of the 2020 calendar year (i.e., 7 months following the end date of Cohort 1′s intended enrollment period). Our explicit goal was to engage UHAND scholars in research, yielding peer-reviewed academic work products such that 2 presentations would be achieved by each undergraduate scholar, 1 presentation and 1 publication would be achieved by each graduate scholar, and 2 presentations and 2 publications would be achieved by the postdoctoral fellow. Goals in excess of this could be pursued by the mentor and mentee as part of the IDP. It is notable that the MDA and the UH PIs were each involved in organizing local conferences to which the scholars could and did submit research for presentation: “Eyes Have Not Seen, Ears Have Not Heard: Breakthroughs in Cancer Research” (Fall 2019), and the “Inaugural HEALTH Research Institute conference” (Winter/Spring 2020). Other presentation opportunities included the annual “Undergraduate Research Day” sponsored by the UH Honors College, and a host of national conferences to which scholars could submit abstracts for presentation.

#### 2.2.3. Program Retention (Goal 3)

Scholar program retention was measured by the comparison of the number of scholars from the time of enrollment to program completion (exit). Our explicit goal was to retain 100% of scholars in the 2-year program by program completion.

#### 2.2.4. Mentee Evaluation of Mentors (Goal 4)

Surveys with questions on scholar evaluations of their mentors were administered at mid-program and at exit. The postdoctoral fellow and graduate scholar responses were combined to facilitate confidentiality. Fleming et al.’s mentor competency assessment was used [37]. This 26-item assessment enables mentees to evaluate 6 mentor competencies in the topic areas of maintaining effective communication, aligning expectations, assessing understanding, fostering independence, addressing diversity, and promoting professional development. Each competency had 2–6 questions asking how skilled the mentee feels the mentor was in the mentioned areas (e.g., active listening, setting research goals, building confidence, etc.) on a scale of not at all (1) to extremely (7). Our explicit goal was to obtain overall scholar ratings of their mentors as at least “moderately” skilled for each mentor competency assessed. The scholar evaluations demonstrated strong reliabilities in all subscales, in both years (2019: 0.91–0.98; 2020: 0.95–1.00).

#### 2.2.5. UHAND Program Feedback and Strategies Learned (Goals 5 and 6)

Exit surveys on program feedback and strategies learned were administered to scholars. The postdoctoral fellow and graduate scholar responses were combined to facilitate confidentiality. Scholars were asked to rate their experiences in the following: 10 categories of UHAND educational seminars and sessions on a 5-point Likert scale, from very poor (0) to excellent (4), with an option to select N/A; UHAND programmatic aspects via 12 items on a 5-point Likert scale from very poor (0) to excellent (4); and general program satisfaction in 5 different programmatic activities on a 5-point Likert scale, from very poor (0) to excellent (4), with an option to select N/A. Face valid, investigator-generated items were used to assess confidence in using strategies learned from UHAND trainings, workshops, and books. These questions asked scholars to rate their confidence in time management, conflict resolution, productivity in writing and completing writing projects, engaging in difficult conversations, career exploration, being resilient in academic rejections, and being resilient in discriminatory/unfair experiences in academia/training on a 5-point Likert scale, from not confident at all (0) to completely confident (4), with an option to select N/A. Our explicit goals were to obtain scholar ratings of the UHAND Program and programming, from “good” to “excellent” (i.e., 3–4), and of confidence in using strategies learned in the UHAND Program, from “fairly confident” to “completely confident” (i.e., 3–4).

#### 2.2.6. Post-Program Progression (Goal 7)

Scholar post-program progression was assessed through surveys that scholars completed monthly during their participation in the program and bi-annually thereafter. Our explicit goal was to have scholars pursue progression in their education and career. Specifically, to have 100% of undergraduate scholars pursue admission to a graduate program (e.g., master’s programs, doctoral programs, medical school), 100% of graduate scholars pursue a postdoctoral training or faculty position, and the postdoctoral fellow obtain a tenure-track faculty position following program completion.

### 2.3. Analyses

Descriptive statistics, including means (and standard deviations, SDs) and frequencies (and percentages), were calculated for continuous (i.e., research self-efficacy, mentee evaluation of mentors, and UHAND Program feedback) and binary variables. To account for the small sample size, the Wilcoxon signed-rank test was used to analyze data between matched subjects for differences in distribution and for outcomes of interest over time. Alpha was set at 0.05. All analyses were conducted using SAS 9.4 [38]. 

## 3. Results

### 3.1. Research Self-Efficacy (Goal 1)

Table 2 presents the means and standard deviations for each of the 14 research self-efficacy items and for the total research self-efficacy score of all scholars across years and by groups (undergraduates vs. graduates/post doc). The means of total research self-efficacy scores across all scholars were 93.4 (SD = 18.75), 88.9 (SD = 27.8), and 106 (SD = 16.63) for 2018, 2019, and 2020, respectively. The total research self-efficacy score for all scholars significantly increased from 2019 to 2020 (88.9 vs. 106, *p* = 0.0273) and from 2018 to 2020 (93.4 vs. 106, *p* = 0.0293), but not from 2018 to 2019 (93.4 vs. 88.9, *p* = 0.5566). Thus, our explicit goal of increasing the research self-efficacy of scholars from baseline (2018) to exit (2020) was achieved. A closer examination by scholar group indicated that the research self-efficacy of undergraduate scholars increased significantly from 2019 to 2020 (74 vs. 102.83, *p* = 0.0313), but not from 2018 to 2019 (83.67 vs. 74, *p* = 0.2188), or from 2018 to 2020 (83.67 vs. 102.83, *p* = 0.0625). No significant differences were found in graduates/post docs in research self-efficacy scores across years.

### 3.2. Academic Products (Goal 2)

Table 3 displays the academic presentation and publication goals for scholars. Regarding academic presentations, 100% of scholars exceeded presentation goals. Regarding publication goals, the postdoctoral fellow, 1 graduate scholar, and 1 undergraduate scholar met or exceeded goals; however, 2 graduate scholars failed to achieve this goal. Thus, only 60% (3/5) achieved/surpassed publication goals. However, it is important to note that 1 of these graduate scholars achieved multiple publications over this time span, though not with her UHAND mentor (not shown in Table 3), and the other graduate scholar has manuscripts in progress with her UHAND mentor (not shown in Table 3). Overall, we partially achieved academic product goals during the assessed time span.

### 3.3. Program Retention (Goal 3)

Five of six undergraduate scholars completed the 2-year program (83.33%), with 1 scholar completing only 1 year due to an opportunity to graduate early. All 3 graduate scholars completed the 2-year program (100%), and the postdoctoral fellow left after a year to take a tenure-track faculty position (0%). Overall, program retention was 80%. As such, we did not achieve our goal to retain 100% of scholars in the 2-year UHAND Program by program completion. However, none of our scholars dropped out of the program while still enrolled in/employed by UH full time; thus, we retained them in the program as long as they remained eligible for support as UHAND scholars (Table 3).

### 3.4. Mentee Evaluation of Mentors (Goal 4)

Descriptive statistics for different aspects of the mentee evaluation of mentors by scholars in the 2019 and 2020 surveys are presented in Table 4. There was no significant difference between 2019 and 2020 on any aspect of the scholar evaluations of mentors. All mean ratings were above the threshold for at least “moderately skilled” (Table 4), indicating that we had achieved this program goal.

### 3.5. UHAND Program Feedback and Strategies Learned (Goals 5 and 6)

Table 5 shows scholar feedback on the quality of UHAND educational seminars and sessions, the quality of other UHAND programmatic aspects, general program satisfaction, and confidence in using strategies learned in the UHAND Program. All ratings were above the scale of 3 (i.e., “good” for UHAND Program seminars/sessions, programmatic aspects, and general program satisfaction; and “fairly confident” for confidence in using the strategies learned) except for 2 ratings: community service-learning experience with community partners (Mean = 2.6, SD = 1.07) and community service-learning experience with UHAND staff/health educators (Mean = 2.78, SD = 1.09). Thus, we failed to achieve our goal of obtaining scholar ratings from “good” to “excellent” (i.e., 3–4) for UHAND programming, and ratings from “fairly confident” to “confident” (i.e., 3–4) for using strategies learned in the UHAND Program, particularly in the case of 2 of 34 (5.89%) UHAND Program seminars/sessions, both in the area of community service-learning.

### 3.6. Post-Program Career Progression (Goal 7)

All 6 undergraduate scholars pursued admission into health science graduate (4 scholars) or medical school (2 scholars) programs (100%), 5 of whom have reported program acceptance to date. It is too early to assess the 3 graduate scholars’ program progression, as they are all currently in good standing in graduate school. The postdoctoral fellow secured a tenure-track faculty position. With follow up, we will be able to evaluate the launch of the 3 graduate scholars in their career progression.

## 4. Discussion

This report described the implementation of the UHAND Program, a UH and MDA collaboration to create a comprehensive career development training program for scholars underrepresented in the fields of behavioral cancer prevention and cancer disparities research, funded by the NCI’s Partnerships to Advance Cancer Health Equity initiative. The UHAND Program incorporated best practices of successful training programs, such as integrated education (diverse professional teams working together, cross-training, exposure to a broad range of disciplines, an integrated curriculum with different subjects, and a variety of learning methods) [29,30,31]; professional development [29]; collaboration between researchers and advocates [24,25]; collaborative partnerships between institutions [32]; broad stakeholder engagement [25,29]; targeted approaches for at-risk groups [25]; training programs at predoctoral, doctoral, and postdoctoral levels [30]; continuous evaluation data on programmatic impact and outcomes [30], while uniquely focusing on research and training in behavioral risk factors for cancer—prioritizing diverse women and minority researchers for training—and facilitating knowledge dissemination and translation between researchers and community. This report presents outcomes relative to the 7 goals of the UHAND Program’s work with scholars and information that may be helpful for the design of future training programs that share similar goals.

The first goal to increase scholars’ research self-efficacy from program baseline to exit was achieved. The UHAND Program provided scholars with research experiences, didactic coursework, career development seminars, and interactive community-based-learning experiences guided through IDPs with their mentors. Scholars were also involved in presenting research to community representatives and audiences, as well as professional/academic audiences. For our undergraduate scholars, they also participated in the SURF program at the UH, which has several didactic research and ethics lectures, tours of labs on campus, and research-building skills over a 10-week summer term. These types of experiences were designed to help build and develop their research self-efficacy skills through mastery via skill-building (e.g., through hands-on mentored research projects), vicarious learning (e.g., through engagement in research seminars given by established researchers), and support (e.g., through fellowship activities, EP check-ins, PI meetings with mentees and mentors). Overall, research self-efficacy significantly increased over the 2-year program, although this was driven by the undergraduate scholars and followed an overall decrease in self-efficacy from the pre-program to the mid-program assessments. The latter may be explained by the Dunning–Kruger Effect, whereby inexperienced people tend to have high confidence and falsely and unknowingly rate their performance highly [39]. Over time and with gained experience, they realize how much they do not know, and this may cause a sharp decline in self-confidence [39]. One report has shown that less competent junior physicians tended to rate their self-efficacy higher than what it was, while competent junior physicians, especially women, tended to rate their self-efficacy lower than those who were less competent [39]. Suggestions for prospective programs would be for the program directors to be aware of the Dunning–Kruger Effect, and particularly that self-efficacy may not translate directly to performance [39]. Tailoring the assessment to the education level of the scholar would be advisable (e.g., undergraduate vs. graduate scholar) by setting clear expectations, measures, and providing factual feedback on skills for undergraduate scholars [39]. Another suggestion is to have assessments and feedback from multiple sources to accurately capture the scholars’ progress in the program [39]. Additionally, identification of research self-efficacy skills that received lower ratings relative to the others could be used to develop programming for future scholars. This may be particularly important for programs that are designed to be less than 2 years in duration, given that there may be a sharp decline in self-efficacy over time that should be monitored and addressed in real-time as the scholars enter the research arena. Moreover, it is also worth noting that the more educated group (graduate scholars and the postdoctoral fellow) did not experience statistically significant increases in self-efficacy over time, in contrast to the undergraduate group. Nevertheless, while self-efficacy ratings were higher among this group of scholars relative to the undergraduates to begin with, ratings generally rose from pre-training to exit (from 108 in 2018 to 110.75 in 2020) and were quite high on average, given that the scale range was from 0 to 126. Their smaller group size and higher starting point relative to the undergraduates may have also affected the inability to achieve statistically significant increases in overall self-efficacy.

It is also worthy of note that overall interest in a cancer disparities career declined over time in the program, and the likelihood of pursuing a career in cancer disparities experienced a mid-program dip, similar to scholar self-efficacy. We believe that this pattern may be similarly explained—greater experience with something may reveal nuances and challenges that dissuade further pursuit and alter original intentions based on new information gained through experience. However, it is important to note that these changes were not statistically significant for any group, sample sizes were very small such that a single rater could significantly influence averages, and all average ratings were ≥3.0 in every year and every scholar group, equivalent to “very interested” and “very likely” to pursue a career in health disparities (except for the undergraduates’ mid-program dip to 2.83). Nevertheless, we failed to completely advance interest and intention to extreme interest/likelihood over time for our scholars; this suggests more work is needed to better understand scholars’ responses and thus develop programming to heighten interest/intention, perhaps by further enhancing self-efficacy, addressing imposter syndrome, and/or focusing on scaffolding approaches that clearly indicate pathways to such careers beyond the training program itself. Qualitative feedback, potentially gathered by a researcher not affiliated with the training program, may be helpful in better understanding these patterns and may be an advisable evaluation method to build into similar programs a priori so that issues can be addressed in real-time, as applicable.

The second goal was to achieve peer-reviewed academic work products amongst the undergraduate, graduate, and post-doctoral scholars. While all scholars achieved the minimum number of presentations, just over half of the scholars achieved publication goals with wide variability amongst scholars (e.g., one undergraduate scholar gave 8 presentations and a graduate scholar gave 11 presentations by mid-program; one undergraduate scholar published 2 empirical manuscripts). Variability in achieving publication goals was at least partially attributable to some scholars working on new projects in the design or data collection phase, whereas others worked with existing data that facilitated faster manuscript development. Additionally, the publication of scientific articles often takes time, which may partially explain why some scholars did not achieve the publication goals during the 2-year program. Overall, results inform approaches for program leadership to take when setting expectations with research mentors, who could in turn set specific publication goals with attainable timelines in scholars’ IDPs. This may include recommendations that mentors provide scholars with access to datasets for secondary analysis and manuscript development while they are involved with ongoing research data collection. Other similar programs could make specific recommendations for academic work product outcomes or provide access to datasets for scholars with mentors who do not have secondary data to work with.

The third goal—attaining complete 2-year program retention—was not achieved, but not for undesirable reasons. Some scholars obtained outstanding career opportunities for which they left the training program early, such as our postdoctoral scholar, who was offered a tenure-track faculty position. In UHAND, applicants to the program were required to have at least two years remaining prior to graduation so that they could participate in the entire 2-year program; however, training programs need to be flexible for changing circumstances or opportunities over time that may affect a scholar’s original plan. It is noteworthy that apart from the postdoctoral scholar who left the program early due to a job offer, all other scholars remained in the program until completion or (unanticipated early) graduation; this positive result partly supports the high program satisfaction expressed by participants. However, it is important to note that UHAND graduate scholars reported becoming overburdened by UHAND program requirements in their second fellowship year, as they also began their required practicum clinical work in the community, which required additional time and travel that affected their availability for UHAND seminars and trainings. Additionally, two of the three graduate scholars in the UHAND Program were paired with a mentor other than their counseling psychology doctoral mentor, which may have increased the pressure on these scholars to conduct research with UHAND in addition to that with their doctoral program mentor. Thus, the design and duration of future training programs should take into account how scholars’ degree program requirements may vary over time, and how the training program can be adapted in kind to ensure academic success and prevent scholar burn out. Additionally, we recommend that graduate scholars in research training programs similar to UHAND be paired with their doctoral mentors for training program research in order to reduce the burden associated with having multiple research mentors.

The fourth goal was to obtain a mentee evaluation of mentors of at least “moderately skilled” in the competencies desired of mentors for the UHAND program, specifically in clear communication, setting expectations, being understanding, fostering independence, respecting diversity, and guiding professional development. We achieved this goal, and thus our program may provide a suitable example for mentor and mentee collaboration. The PIs of the UHAND Program were both department chairs at their respective institutions and had a deep understanding of the faculty across departments doing work in cancer disparities. As the PIs personally knew the mentors and/or supervised them in these roles, they were able to successfully match them with scholars and guide them in this process. Generally, we recommend that other training programs work with individuals who have vast institutional knowledge/experience/connections when selecting mentors for program inclusion. In the absence of such knowledge, or perhaps in any case, programming could also be provided to support and develop the mentors in the execution of their roles. In the UHAND program, we routinely communicated with the mentors about external opportunities (e.g., locally held workshops) to enhance their mentorship skills, but we offered no formal training ourselves nor did we assess or monitor training that they may have received. In retrospect, offering and assessing the results of mentor training may have further enhanced competencies in this area and might be particularly appropriate for enhancing training with women and minority scholars, who may not be of the same sex/ethnicity/race as their mentors.

The fifth and sixth goals were to achieve ratings from “good” to “excellent” on UHAND Program Feedback, and ratings from “fairly” to “completely confident” on strategies learned by our scholars. The programming included a range of seminars designed to enhance time management, facilitate competency in academic writing, explore career options in cancer disparities, and develop skills in presenting oneself and one’s research in both academic and community settings. “Good” to “excellent” scores were achieved for each program element, with the exception of the two tapping into community service-learning experiences. Feedback provided by the scholars indicated that they were, at times, assigned tasks at community agencies that were rather mundane (e.g., filing), which contributed to dissatisfaction. Feedback from the community agencies in which scholars were placed indicated that they were used to working with more substantial scholar time commitments than what this portion of the UHAND Program allowed (i.e., about 30 h a semester) and thus struggled with how to involve our scholars in substantive and meaningful work. Hence, training programs with service-learning components should explore methods that would enable more time at community agencies, particularly in ways that do not increase overall programmatic time requirements. To give an example, this might be achieved by assisting scholars to get course credit for work with community agencies (e.g., internship experiences) or degree-required clinical practice hours in these settings. Despite challenges, program satisfaction was high, and scholars indicated they were “fairly” to “completely confident” in using many of the strategies and skills they were taught in practice (e.g., engaging in difficult conversations, being resilient in handling discriminatory or unfair experiences in the academy).

The seventh goal was to track post-program progression, with undergraduate scholars pursuing graduate program admissions, graduate scholars pursuing postdoctoral or faculty positions, and the post-doctoral scholar obtaining a tenure-track faculty position. We achieved this goal with the post-doctoral and undergraduate scholars, the latter of whom went to a combination of master’s degree, doctoral degree, or medical degree programs. It is too early to assess the 3 graduate scholar’s program progressions as they remain in good standing in graduate school. With an ultimate program goal of supporting scholars in pursuit of careers in cancer disparities research, it is important to have programs build processes for continuous monitoring and guidance of scholar achievements that extend beyond graduation and the program funding period. To this end, we will continue to follow our scholars over time to track their academic career achievements and ultimate employment as trained professionals. Mechanisms to bolster continued engagement with scholars over time (e.g., periodic newsletters and mailings, “reunion” dinners or virtual events) will need to be considered.

While the UHAND Program achieved many goals, lessons were learned that could enhance similar training programs in the future. Although the UHAND Program represents a model for educational training programs focused on reducing health disparities, particularly those focused on cancer, there are limitations that need to be considered in the evaluation of our goals. These include the fact that lessons may not generalize to other cohorts, institutional collaborations, or mentors. This may especially be the case given the low representation of men (n = 1) in our initial cohort of scholars. Future programs may wish to determine a desired sex distribution a priori and select scholars accordingly, inasmuch as a balance between the sexes is desirable. Additionally, all the graduate scholars were in counseling psychology, which is likely a reflection of the UH PI’s affiliation with that program and the program’s focus on health-oriented, community-engaged work and social justice, which complements the UHAND mission. However, the UHAND Program serves as a model of a grant-supported training program that spanned three diverse educational levels at a minority serving institution, achieved excellent collaboration with a comprehensive cancer center that gave scholars access to many opportunities unavailable at their home institution, and provided diverse scholar access to the cancer center faculty for research training.

## 5. Conclusions

The UHAND Program may inform other educational training programs that aim to reduce inequities in cancer, and in health more broadly, by increasing the number of underrepresented racial/ethnic minority student scholars through the provision of research training, mentorship, and service-learning opportunities. The UHAND Program also provides an example of successful engagement and collaboration between institutions, and with community via community outreach. On the whole, the UHAND Program may provide a comprehensive, evidence, and need-based model for other educational training programs with similar goals.

## Figures and Tables

**Table 1:** UHAND Program Participants, Cohort 1 (N = 10).

Scholar	Disadvantaged Background ^†^	First Generation College *	Female Sex	Ethnicity/Race	University Major
Postdoc	X		X	Black	N/A
Grad 1	X		X	Black	Counseling Psychology
Grad 2		X	X	non-Hispanic White	Counseling Psychology
Grad 3			X	non-Hispanic White	Counseling Psychology
UG 1	X	X		Black	Health
UG 2			X	Asian American	Biology
UG 3			X	Hispanic White	Psychology
UG 4	X		X	Black	Health
UG 5			X	Black and non-Hispanic White	Biochemical/Biophysical Science
UG 6	X	X	X	Hispanic White	Health

Note: ^†^ Underrepresented racial and ethnic groups in health-related sciences, individuals with physical or mental disabilities, individuals from low-income families, and individuals from inhibiting educational environments [19]; * Scholars who are the first generation from their families to attend college; Postdoc: Postdoctoral Fellow; Grad: Graduate Scholar; UG: Undergraduate Scholar.

**Table 2:** Research Self-Efficacy and Cancer Career Interest by Scholar Groups and Year (N = 10).

	2018 ^a^			2019 ^b^			2020 ^c^		
	All (n = 10) ^1^	UG ^2^ (n = 6)	Grad/PD (n = 4)	All (n = 10) ^1^	UG ^2^ (n = 6)	Grad/PD (n = 4)	All (n = 10) ^1^	UG ^2^ (n = 6)	Grad/PD (n = 4)
	Mean (SD)								
Selecting a suitable topic for a study	5.10 (2.38)	3.83 (2.04)	7.00 (1.41)	6.60 (2.8)	5.33 (3.01)	8.50 (0.58)	8.30 (0.67)	8.5 (0.55)	8.00 (0.82)
Knowing which stats to use	4.40 (3.17)	2.33 (2.25)	7.50 (0.58)	4.80 (3.05)	4.00 (2.83)	6.00 (3.37)	6.30 (2.63)	5.83 (3.13)	7.00 (1.83)
Getting adequate number of participants	6.30 (1.77)	6.00 (1.67)	6.75 (2.06)	6.70 (2.36)	5.67 (2.58)	8.25 (0.5)	8.10 (0.88)	8.33 (0.82)	7.75 (0.96)
Writing a research presentation for conference	6.30 (1.83)	5.33 (1.51)	7.75 (1.26)	6.70 (2.41)	5.50 (2.43)	8.50 (0.58)	8.20 (0.92)	8.00 (1.10)	8.50 (0.58)
Writing a research paper	7.30 (1.25)	7.17 (1.33)	7.50 (1.29)	6.60 (2.59)	5.67 (3.08)	8.00 (0.00)	7.90 (0.88)	7.67 (1.03)	8.25 (0.50)
Collecting data	7.10 (1.73)	6.67 (2.07)	7.75 (0.96)	6.90 (2.56)	6.17 (3.13)	8.00 (0.82)	7.40 (2.67)	7.00 (3.46)	8.00 (0.82)
Making time for research	8.00 (1.05)	8.17 (1.17)	7.75 (0.96)	7.10 (2.13)	6.33 (2.42)	8.25 (0.96)	8.10 (0.74)	8.50 (0.55)	7.50 (0.58)
Reviewing the literature in an area of research interest	8.10 (1.20)	7.83 (1.33)	8.50 (1.00)	7.50 (2.07)	7.00 (2.53)	8.25 (0.96)	8.60 (0.70)	8.67 (0.82)	8.50 (0.58)
Contacting researchers currently working in an area of research interest	7.40 (1.51)	7.00 (1.79)	8.00 (0.82)	7.30 (1.83)	6.50 (1.97)	8.50 (0.58)	7.90 (1.20)	7.67 (1.51)	8.25 (0.50)
Formulating hypotheses	7.30 (1.49)	6.67 (1.51)	8.25 (0.96)	6.60 (1.96)	5.83 (2.23)	7.75 (0.50)	7.30 (1.49)	7.33 (1.97)	7.25 (0.50)
Operationalizing variables of interest	5.90 (2.81)	4.50 (2.74)	8.00 (1.15)	5.30 (2.98)	3.67 (2.8)	7.75 (0.50)	6.80 (2.94)	6.00 (3.69)	8.00 (0.00)
Choosing research design that will answer research question or test hypotheses	5.90 (2.02)	4.83 (1.47)	7.50 (1.73)	5.60 (2.91)	4.17 (2.99)	7.75 (0.50)	6.20 (2.82)	5.33 (3.39)	7.50 (1.00)
Apply appropriate process for obtaining informed consent from subjects	7.40 (1.35)	7.00 (1.41)	8.00 (1.15)	6.10 (2.56)	4.83 (2.56)	8.00 (0.82)	7.80 (1.32)	7.50 (1.64)	8.25 (0.50)
Write human subjects consent form containing appropriate elements	6.90 (1.73)	6.33 (1.75)	7.75 (1.50)	5.10 (2.92)	3.33 (2.42)	7.75 (0.50)	7.10 (1.52)	6.50 (1.64)	8.00 (0.82)
Total Research Self-Efficacy (possible range: 0 to 126) ^1ac*; 1bc*; 2bc*^	93.40 (18.75)	83.67 (16.15)	108.00 (12.11)	88.90 (27.80)	74.00 (26.91)	111.25 (1.26)	106.00 (16.63)	102.83 (21.25)	110.75 (5.19)
Cancer Career Interest									
How interested are you in pursuing a career in cancer disparities research? ^†^	3.70 (0.48)	3.67 (0.52)	3.75 (0.50)	3.50 (0.85)	3.33 (1.03)	3.75 (0.50)	3.30 (0.82)	3.33 (0.82)	3.25 (0.96)
How likely is it that you will pursue a career in cancer disparities research? ^†^	3.40 (0.70)	3.33 (0.82)	3.50 (0.58)	3.20 (1.03)	2.83 (1.17)	3.75 (0.50)	3.30 (0.67)	3.50 (0.55)	3.00 (0.82)

Note: ^a^ Comparisons involving 2018 (baseline) data; ^b^ Comparisons involving 2019 (mid-program) data; and ^c^ Comparisons involving 2020 (exit) data. ^1^ Comparisons involving all scholars; ^2^ Comparisons involving undergraduate scholars. Item scores range from 0 (No confidence) to 9 (Total confidence); ^†^ 0 (Not at all)–4 (Extremely); * *p* < 0.05; UG: Undergraduate Scholar; Grad/PD: Graduate Scholars and Postdoctoral Fellow. The significance of findings for Grad/PD analyses were unchanged when the PD data were removed from the dataset.

**Table 3:** UHAND Scholars’ Academic Products ^†^ and Program Retention, Cohort 1 (N = 10).

Scholar	Duration in Program (in Years)	Academic Presentation Goals	Presentations Given (% at Regional, National, or International Outlets)	Peer-Reviewed Publication Goals	Number of Peer-Reviewed Publications Achieved
Postdoc	1	2	6 (50.0%) **	2	6 **
Grad 1	2	1	8 (62.4%) **	1	0
Grad 2	2	1	17 (52.9%) **	1	1 *
Grad 3	2	1	8 (87.5%) **	1	0
UG 1	2	2	4 (50.0%) **	N/A	2 **
UG 2	2	2	13 (30.8%) **	N/A	N/A
UG 3	2	2	3 (66.7%) **	N/A	N/A
UG 4	1	2	4 (75.0%) **	N/A	N/A
UG 5	2	2	3 (33.3%) **	N/A	N/A
UG 6	2	2	5 (80.0%) **	N/A	N/A

Note: ^†^ Reflects academic work products acknowledging UHAND support. Please note that 6 of the 71 presentations listed featured 2 UHAND scholars as co-authors; thus, there were 65 total unique presentations. Peer-reviewed publications include accepted manuscripts, manuscripts in press, and paginated publications. Postdoc: Postdoctoral Fellow; Grad: Graduate Scholar; UG: Undergraduate Scholar; * Met goal; ** Surpassed goal.

**Table 4:** Scholar (N = 10) Evaluation of Mentors (N = 11) ^†^.

How Skilled YOU feel Your Mentor Is in the Area of…..	Number of items (Potential Range)	2019	2020	Threshold for ≥ “Moderately Skilled”
		Mean (SD)		
Maintaining Effective Communication	6 (6–42)	35.80 (6.05)	34.30 (9.67)	≥24
Aligning Expectations	5 (5–35)	30.80 (4.52)	29.20 (8.87)	≥20
Assessing Understanding	3 (3–21)	17.44 (3.40)	17.30 (5.31)	≥12
Fostering Independence	5 (5–35)	29.00 (6.88)	30.10 (8.77)	≥20
Addressing Diversity	2 (2–14)	12.22 (2.39)	11.60 (3.86)	≥8
Promoting Professional Development	5 (5–35)	28.80 (6.99)	27.80 (8.07)	≥20

Note: Responses for each item ranged from 1 to 7, where 1 = Not at all; 4 = Moderately; and 7 = Extremely. ^†^ A total of 11 mentor-mentee dyads were assessed across 10 scholars; 1 undergraduate scholar switched mentors after Year 1 (and thus had 2 mentors).

**Table 5:** UHAND Program Feedback and Strategies Learned from UHAND Programming.

Aspects	Items	Mean (SD)
Scholars’ Feedback on UHAND Educational Seminars and Sessions ^a^	POWER Writing	3.44 (0.53)
	Elevator Speech Workshop	3.38 (0.52)
	Time Management Workshop	3.25 (0.71)
	What Color is Your Parachute Workshop	3.33 (0.50)
	UH Summer Undergraduate Research Fellowship (SURF) ^†^	3.67 (0.82)
	UHAND Cancer Disparities Seminar	3.60 (0.52)
	Grand Rounds/Brown Bag Series	3.20 (1.32)
	Laboratory Research Experience	3.25 (0.89)
	Community Service-Learning Experience with Community Partners	2.60 (1.07)
	Community Service-Learning Experience with UHAND Staff/Health Educators	2.78 (1.09)
Scholars’ Feedback on UHAND Programmatic Aspects ^a^	Orientation	3.70 (0.48)
	Help from UHAND staff and faculty members	3.90 (0.32)
	Communication from UHAND	3.60 (0.52)
	Processes and procedures (e.g., for reimbursement, timesheets)	3.50 (0.71)
	Stipend or hourly pay rate received	3.40 (0.70)
	Other resources received (e.g., books)	3.60 (0.70)
	Frequency of meetings with your research mentor	3.40 (0.84)
	Quality of communication with your research mentor	3.40 (0.84)
	Quality of meetings with your research mentor	3.30 (0.82)
	Quality of the guidance received from your research mentor	3.40 (0.84)
	Quality of the overall experience with your research mentor	3.30 (1.06)
	Quality of the research experiences offered to you by your research mentor	3.40 (0.97)
General Program Satisfaction ^b^	I am satisfied with my overall experience as a UHAND Scholar.	3.70 (0.48)
	I felt supported as a UHAND Scholar.	3.80 (0.42)
	I have increased my ability to be an independent researcher as the result of UHAND.	3.60 (0.52)
	The UHAND Program was important to my career development.	3.70 (0.48)
	Programs like UHAND are necessary for diversifying the pipeline of scholars going into the cancer disparities field.	4.00 (0.00)
Scholars’ Confidence using Taught Strategies ^c^	Time management	3.22 (0.67)
	Conflict resolution	3.40 (0.52)
	Productivity in writing and completing writing projects	3.40 (0.52)
	Engaging in difficult conversations	3.33 (0.50)
	Career exploration (from What Color is Your Parachute workshops)	3.22 (0.67)
	Being resilient in your handling of academic “rejections”	3.22 (0.44)
	Being resilient in your handling of discriminatory or unfair experiences in academia/training	3.44 (0.53)

Note: ^†^ This is applicable only to undergraduate scholars; ^a^ The item scale is from 0 to 4, where 0 = Very poor; 1 = Poor; 2 = Average; 3 = Good; and 4 = Excellent; ^b^ The item scale is from 0 to 4, where 0 = Strongly disagree; 1 = Disagree; 2 = Neither agree nor disagree; 3 = Agree; and 4 = Strongly agree; ^c^ The item scale is from 0 to 4, where 0 = Not confident at all; 1 = Slightly confident; 2 = Somewhat confident; 3 = Fairly confident; and 4 = Completely confident.

## Data Availability

The data presented in this study are available on request from the corresponding author. The data are not publicly available due to privacy and confidentiality concerns given the very small group of mentees and mentors and the ability to link the two from the data alone, which could affect dynamics of ongoing mentoring relationships in unknown ways.
